# Barriers and facilitators to implementation of VA home-based primary care on American Indian reservations: a qualitative multi-case study

**DOI:** 10.1186/s13012-017-0632-6

**Published:** 2017-09-02

**Authors:** B. Josea Kramer, Sarah D. Cote, Diane I. Lee, Beth Creekmur, Debra Saliba

**Affiliations:** 1 VA Greater Los Angeles Healthcare System, Geriatric Research Education and Clinical Center, 16111, St (11E), North Hills, Plummer, CA 91343 USA; 2 David Geffen School of Medicine at UCLA, Division of Geriatric Medicine, 10945 Le Conte Avenue, Suite 2339, Los Angeles, CA 90095 USA; 3 Rio Hondo College, Institutional Research & Planning, 3600 Workman Mill Road, Whittier, CA 90601 USA; 4 Kaiser Permanente Research, Department of Research and Evaluation, 100 South Los Robles, Pasadena, CA 91101 USA; 5 UCLA/Jewish Home Borun Center for Gerontological Research, 10945 LeConte Ave, Suite 2339, Los Angeles, CA 90095 USA; 6 RAND Corporation, 1776 Main Street, Santa Monica, CA 90401 USA

**Keywords:** Rural, Veterans, Non-institutional long-term care, Indians, North America, Consolidated Framework for Implementation Research, CFIR

## Abstract

**Background:**

Veterans Health Affairs (VA) home-based primary care (HBPC) is an evidence-based interdisciplinary approach to non-institutional long-term care that was developed in urban settings to provide longitudinal care for vulnerable older patients. Under the authority of a Memorandum of Understanding between VA and Indian Health Service (IHS) to improve access to healthcare, 14 VA medical centers (VAMC) independently initiated plans to expand HBPC programs to rural American Indian reservations and 12 VAMC successfully implemented programs. The purpose of this study is to describe barriers and facilitators to implementation in rural Native communities with the aim of informing planners and policy-makers for future program expansions.

**Methods:**

A qualitative comparative case study approach was used, treating each of the 14 VAMC as a case. Using the Consolidated Framework for Implementation Research (CFIR) to inform an open-ended interview guide, telephone interviews (*n* = 37) were conducted with HBPC staff and clinicians and local/regional managers, who participated or oversaw implementation. The interviews were transcribed, coded, and then analyzed using CFIR domains and constructs to describe and compare experiences and to identify facilitators, barriers, and adaptations that emerged in common across VAMC and HBPC programs.

**Results:**

There was considerable variation in local contexts across VAMC. Nevertheless, implementation was typically facilitated by key individuals who were able to build trust and faith in VA healthcare among American Indian communities. Policy promoted clinical collaboration but collaborations generally occurred on an ad hoc basis between VA and IHS clinicians to optimize patient resources. All programs required some adaptations to address barriers in rural areas, such as distances, caseloads, or delays in hiring additional clinicians. VA funding opportunities facilitated expansion and sustainment of these programs.

**Conclusions:**

Since program expansion is a responsibility of the HBPC program director, there is little sharing of lessons learned across VA facilities. Opportunities for shared learning would benefit federal healthcare organizations to expand other medical services to additional American Indian communities and other rural and underserved communities, as well as to coordinate with other healthcare organizations. The CFIR structure was an effective analytic tool to compare programs addressing multiple inner and outer settings.

## Background

Little is known about dissemination of home-based primary care (HBPC) to rural communities and, in particular, rural American Indian reservations. HBPC is a non-institutional long-term care program that provides ongoing comprehensive, interdisciplinary primary care to patients in their homes when healthcare can no longer be optimally provided in a clinic setting. By this definition, the Agency for Health Research and Quality [1] found only 19 studies that report on the outcomes of these programs: one in Canada, two in Denmark, and 16 in the USA, with 50% of US programs delivered by Veterans Health Affairs (VA) [2–9]. Recently, 14 VA medical centers (VAMC) began expansion of the evidence-based HBPC program to reach new populations of American Indian veterans living in rural reservation communities, which are served by the Indian Health Service (IHS) or Tribal Health Programs (THP) [10]. Each VAMC independently developed strategies and models to implement their rural HBPC programs and we treat each as a case study for comparative analysis. Two HBPC programs were unable to establish programs in these communities, while 12 succeeded, leading to our study question: what are the barriers and facilitators for implementation of a new clinical program on rural American Indian reservations. Secondarily, we aim to describe how two healthcare organizations work together to optimize healthcare resources as a result of implementing the new program. Our goal is to inform planners and policymakers about factors that may facilitate or hinder successful programs in underserved communities.

VA and IHS are US federal healthcare programs that have different eligibility requirements. VA medical care is an entitlement for veterans who meet basic or enhanced eligibility requirements that consider length of military service, period of service in war or declared conflict, income, and service-connected disability or illness [11]. Veterans are assigned to “priority groups,” which are based on factors such as the extent of service-connected injuries or illnesses and on personal income; the priority group determines if VA services are delivered at no cost or if the veterans must assume a co-pay fee to cover costs. IHS, an agency within the US Department of Health and Human Services, provides health services for 567 federally recognized sovereign Tribes [12]. IHS is not an entitlement program and eligibility is determined by Tribal membership, residence, and location of services [13]. Tribes may accept direct care from IHS or may administer IHS funding within their own tribal health programs (THP). Some American Indian and Native Alaska veterans are eligible for both VA and IHS/THP and co-managed care or dual use allows patients to “mix and match” services across healthcare organizations [14, 15]. American Indian veterans may travel off-reservation to a VAMC or VA community community-based outpatient clinic to receive healthcare. Any VA presence in a reservation community must be authorized by the sovereign Tribe, including clinical programs, outreach activities to describe veteran benefits, and no-cost “health fairs” to screen community members for chronic diseases and provide health education.

Collaboration between VA and IHS is supported by VA strategic plans [16], interagency agreements, and national policy [17] that calls for meaningful consultation and collaboration by federal agencies with Tribes. In 2010, VA and IHS executed a Memorandum of Understanding with the specific goal “to improve the delivery of care through active sharing of care processes, programs and/or services with benefit to those served by both IHS and VA.” The Memorandum of Understanding lists HBPC expansion as an example of a shared beneficial program and that expansion continues to be cited in annual progress reports to Congress [18].

HBPC was developed and evaluated in randomized control trials in the 1990s [19–21] and continues to be cost-effective [3, 4] by reducing unnecessary utilization of hospitals or emergency departments without shifting costs to Medicare [5, 22, 23]. The program has been established at over 140 of the 168 VAMC. A VA HBPC Handbook provides detailed guidance for operations under a HBPC program director and clinical supervision by a medical director. HBPC is a complex intervention using a team-based interdisciplinary approach to deliver primary care services with at least 7–10 home and telehealth visits per year. Interdisciplinary team case conferences are conducted weekly and each patient’s management plan is reviewed at least every 90 days. Originally developed for urban VAMC, caseloads average 20–30 for a registered nurse and a maximum of 34 patients for a nurse practitioner. The program is supported financially by the Veterans Equitable Resource Allocation budgeting formula that accounts for its intensive workload in allocating funds to VAMC.

As the largest integrated and open access healthcare system in the USA, VA has long been concerned about the inequities in its healthcare system. Older veterans are more likely than non-veterans to have functional limitations that are associated with increased risk of poor health outcomes [24, 25], more likely to live in rural areas, and less likely to have age-related healthcare needs adequately met [26–31]; these inequities are particularly great for American Indian veterans [32–36]. To address the gap in access to non-institutional long-term care, the VA Office of Rural Health provided seed funding to expand urban HBPC programs to American Indian reservations and to other rural areas [9]. Fourteen VAMC, which were located in eight geographically dispersed regions, expressed interest in expansion and all were funded for 2 years of start-up costs with the expectation that VAMC would later sustain the program through its annual funding allocations.

## Methods

HBPC expansion programs serve as a natural laboratory to understand the interacting and multi-level factors that impede and facilitate implementation. We used a qualitative observational design to retrospectively document expansion and implementation of HBPC on American Indian reservations through key respondent interviews, which were conducted after the programs were fully implemented. Each HBPC program was considered as a case study for comparative analyses [37, 38] to determine if similar issues arose in multiple contexts.

The adage, “if you’ve seen one VAMC, you’ve seen one VAMC” implies the difficulty of studying non-static implementation across multiple settings and requires an efficient analytic structure to identify and describe factors that may influence successful implementation of an intervention in different community settings. The Consolidated Framework for Implementation Research (CFIR) [39, 40] uses a comprehensive multi-level determinant framework to systematically identify barriers and facilitators in five inter-related domains: Intervention characteristics, Outer setting, Inner setting, Characteristics of individuals, and Process. These domains are further defined by 39 pragmatic constructs that reflect theories and hypotheses in organizational and implementation research. CFIR has advantages over frameworks that focus on clinical evidence, guidelines, or innovative program characteristics and, for this study, an additional advantage to CFIR is the flexibility to address complex interactions within the internal VA settings, as well as with external settings of Tribes, IHS/THP, and Native communities. CFIR domains informed the main topical areas of our semi-structured, open-interview guide and prompts were used, as needed, to explore CFIR constructs.

The study sample was structured to represent each of the 14 facilities, as well as levels of responsibility for planning and/or implementing HBPC expansion. Key respondents were selected from lists of knowledgeable persons that were requested of each Chief of Staff. The lists included HBPC administrative and clinical staff (e.g., program director, program coordinator, medical director, primary care provider, social worker), and management leadership with oversight of the HBPC program at the facility (e.g., Chief of Staff, Geriatrics/Extended Care line manager) or regional level (e.g., rural health coordinator, minority Veteran coordinator). Participation was voluntary and Chiefs of Staff were not informed about the identities of volunteer respondents. The final sample of 37 respondents included 20 HBPC clinicians and 17 managers.

Data were collected in 1:1 telephone interviews, which were recorded with respondents’ permissions, transcribed, coded by members of the study team (JK, SC, DL), and entered into Atlas-ti™ software [41]. Coding was an iterative process. Two coders (SC, DL) initially coded narrative text, to identify and classify descriptions within the 39 CFIR constructs. Discordant coding was identified and consensus strategies were used to manage disagreements and refine code definitions as needed to be relevant to this study; if consensus was not achieved, the PI (JK) resolved the issue and provided additional training. As coding definitions were further refined, all three coders then re-coded texts using the operational definitions shown in Table 1. The study was approved by the Institutional Review Board at the VA Greater Los Angeles Healthcare System.

**Table 1:** Description and operational definitions of constructs in the Consolidated Framework for Implementation Research

CFIR Domain and Construct	Brief CFIR Definition^a^	Operational Definition
I. INTERVENTION CHARACTERISTICS		
Complexity	Perceived difficulty of implementation, reflected by duration, scope, radicalness, disruptiveness, centrality, and intricacy and number of steps required to implement.	Challenges, expected and unexpected, to implementing the HBPC pilot
Cost	Costs of the intervention and costs associated with implementing the intervention including investment, supply, and opportunity costs.	Financial costs of the program affecting the decision to implement, the initial plan for implementation, and/or program sustainability
II. OUTER SETTING		
Patient Needs & Resources	The extent to which patient needs, as well as barriers and facilitators to meet those needs, are accurately known and prioritized by the organization.	Knowledge of 1) American Indian patients’ medical needs and eligibility for VA, IHS/THP services, 2) IHS/THP and other regional health resources
Cosmopolitanism	The degree to which an organization is networked with other external organizations.	Relationship and clinical collaborations between VAMC and IHS/THP
External Policy & Incentives	A broad construct that includes external strategies to spread interventions, including policy and regulations (governmental or other central entity), external mandates, recommendations and guidelines, pay-for-performance, collaborative, and public or benchmark reporting.	Policies and incentives that impacted HBPC implementation
III. INNER SETTING		
Structural Characteristics	The social architecture, age, maturity, and size of an organization.	Organizational characteristics of HBPC
Networks & Communications	The nature and quality of webs of social networks and the nature and quality of formal and informal communications within an organization.	Sharing of patient in HBPC interdisciplinary team and other communications, such as referrals, within VAMC
Implementation Climate	The absorptive capacity for change, shared receptivity of involved individuals to an intervention, and the extent to which use of that intervention will be rewarded, supported, and expected within their organization, including the subconstructs of Tension for change, Compatibility, Relative Priority, Organizational incentives and rewards, Goals and feedback and Learning climate.	The degree of compatibility (i.e., tangible fit) between meaning and values attached to the intervention by involved individuals, how those align with individuals’ own norms, values, and perceived risks and needs, and how the intervention fits with existing workflows and systems.
IV. CHARACTERISTICS OF INDIVIDUALS		
Knowledge & Beliefs about the Intervention	Individuals’ attitudes toward and value placed on the intervention as well as familiarity with facts, truths, and principles related to the intervention.	Opinions about HBPC
Other Personal Attributes	A broad construct to include other personal traits such as tolerance of ambiguity, intellectual ability, motivation, values, competence, capacity, and learning style.	Personal traits of individuals involved in HBPC implementation
V. PROCESS		
Executing	Carrying out or accomplishing the implementation according to plan.	Roles of VAMC, IHS/THP in identifying potential patients and delivering services
Reflecting	Quantitative and qualitative feedback about the progress and quality of implementation accompanied with regular personal and team debriefing about progress and experience.	Lessons learned and recommendations

^a^Consolidated Framework for Implementation Research. CFIR Constructs. Available at: http://cfirguide.org/constructs.html. Accessed March 28, 2016

## Results

Each of the VAMC in this study had well-established, mature urban HBPC programs, which continued under the direction of their respective medical directors as they adapted to delivering care at a distance from the VAMC. Few formal relationships with IHS/THP facilities were in place prior to the HBPC expansion, to the best of the knowledge of the study respondents. As indicated in Table 2, local contexts for target populations varied, by size (ranging from <5000 to >100,000 active users), distance (ranging from <50 miles to >200 miles, one-way between health centers and possibly farther to patients’ homes) and on-reservation healthcare system (IHS or THP).

**Table 2:** Variation in target populations for 12 VA medical centers that expanded home-based primary care to rural American Indian reservations

	VAMC 1	VAMC 2	VAMC 3	VAMC 4	VAMC 5	VAMC 6	VAMC 7	VAMC 8	VAMC 9	VAMC 10	VAMC 11	VAMC 12
Population served by:												
IHS					x	x	x			x	x	x
THP		x	x	x				x				
Population: Multiple Tribes	x			x				x	x		x	
Active IHS/THP users at initiation of HBPC expansion^a^	<5000^b^	10,000–30,000	5000–10,000	<5000	10,000–30,000	<5000	<5000	<5000	5000–10,000	>100,000	>100,000	10,000–30,000
Distance in miles from VAMC to furthest IHS/THP clinics in HBPC catchment area	<50	50–100	50–100	>200	100–200	50–100	100–200	50–100	100–200	100–200	>200	50–100
Existing clinical relationship between VAMC and IHS/THP (e.g., cost sharing, joint privileging)		x							x			x

^a^Healthcare Patient Information from Department of Health & Human Services Final User Population Estimates 2010 Report [32]

^b^Tribes not serviced by IHS or THP

There were consistent similarities in challenges to be overcome, barriers that could not be addressed at the program level and facilitators across programs as shown in Table 3.

**Table 3:** Key challenges, barriers and facilitators to expansion of HBPC across 12 VA Medical Centers

	VAMC 1	VAMC 2	VAMC 3	VAMC 4	VAMC 5	VAMC 6	VAMC 7	VAMC 8	VAMC 9	VAMC 10	VAMC 11	VAMC 12
CHALLENGES												
Target population eligibility and need for HBPC unknown	X	X	X	X	X	X	X	X	X	X	X	X
Distance & other rural conditions (e.g., connectivity)	X	X	X	X	X	X	X	X	X	X	X	X
Hiring: recruitment and delays	X	X	X	X		X	X		X		X	X
Patients may have co-pay to use HBPC and other VA services		X	X	X	X	X	X		X	X		X
BARRIERS												
Remote areas of reservation too distant					X					X	X	
Potential patients do not meet VHA medical benefit eligibility		X			X							
FACILITATORS												
Established mature HBPC program, standardized outcome measures and local VAMC referral patterns	X	X	X	X	X	X	X	X	X	X	X	X
Outreach activities to enroll American Indian veterans for VA benefits and/or explain HBPC service	X	X	X	X	X	X	X	X	X	X	X	X
Seed and sustainment funding for expansion	X	X	X	X		X	X	X	X	X	X	X
Personal characteristics of HBPC program staff		X	X		X				X	X	X	X
American Indian community advocate	X	X	X				X					X
Formal or informal referral mechanism for HBPC referral with IHS/THP		X			X		X		X			X

While all CFIR domains were relevant, only 12 of the possible 39 CFIR constructs emerged from the rich text of these interviews. Table 4 represents the implementation experience from the VA perspective in selected quotations. Within each domain, local contexts may be essential to explain variation across cases and we note relevant differences by domain in summary descriptions of cross-case barriers and facilitators below.

**Table 4:** Selected interview quotations on experiences and perceptions of Key Respondents in implementing HBPC on American Indian reservations, organized by CFIR domains and constructs and identifying respondent by HBPC as staff, clinician or VA leadership roles and by an anonymized facility identifier

DOMAIN & Construct	Themes	Representative Quotation from Key Respondent Interviews
INTERVENTION Complexity	Difficulty of working in rural areas: a) Hiring	“The biggest challenge … has been hiring. It’s really difficult to get good quality providers to go work in these rural areas. We get people in, and they’ll come and stay for a little while, and then they’ll move on somewhere else. It’s really difficult to keep good providers.” *Leadership (2)* “[I]t’s hard to find people that want to live in a rural area and work in a rural area. Because most of our tribal entities tend to be a lot further than an hour away from a medical center. And it’s hard to get staff who want to live in that general area that want to do the Home Based Primary Care.” *Leadership (12)*
	b) Distance and location	“The other issue to consider as well is that a number of reservations are very isolated. You’re talking about potentially huge tracts of land… it would take them forever to get there, to find this person in their home. …. I think that’s a really big barrier, is the fact that these reservations typically are very isolated. *Leadership (9)* “[Programs should] factor in [the fact that you’ll]… drive two hours to someone’s house. How many people can you see in a day when you’re taking two hours? So don’t try to overdo it… leave enough time to go, and to be with that Veteran as long as you need to be there, because you don’t want to have to go back.” *HBPC Clinician (12)* “Everybody up there is on a P.O. Box… there are no physical addresses. And that can be a real challenge. I mean, if you have somebody who has difficulty traveling, you have to get a relative to meet you at whatever dirt road turnoff and follow you in.” *HBPC Staff (11)*
	c) Reduced case load	“We hired a second nurse practitioner and a second RN, and… we had originally thought was that they could, between those two, they could case-manage 45 patients and it just turned out that that wasn’t really true. So the RN in particular just was drowning and said, “Really, I cannot manage more than 22 patients,” *HBPC Staff (2)* One of the challenges that we’ve had is a limited number of patients that we’ve been able to enroll on the program because of the extreme distance that we drive to see these patients and because every person on this program has a very significant collateral role with the team—so it really limits our ability to increase our numbers. *HBPC Staff (12)*
Cost	Sustainment potential	“The tribe is actually a fairly small percentage of the Veterans that we serve… never more than 20% have been Native.” *HBPC Clinician (7)* “[The proportion of American Indian patients is] fairly low, I’m thinking about 20%… the [total] census lists anywhere from 40 to 50 so we had… 10 or so Native Americans at any one time serviced. “*HBPC Staff (8)* “[T]here’s a lot of little communities that are scattered all over…but I would tend to say [the percentage of Native patients is] over 50%. *HBPC Clinician (10)*
OUTER SETTING: Cosmopolitan	Collaboration between VA and IHS/THP	“At [THP], if the social worker has a particular veteran that she knows will be getting equipment for the VA, the social worker will give us a call and kind of get an idea of what VA is providing in the home so they won’t duplicate any equipment or stuff.” *HBPC Staff (3)* “A lot of our Home Based Primary Care program veterans that are enrolled in our program actually have primary care locally. So there’s a lot of time and coordination needed to get results from the local hospital or local physician or local specialist.” *HBPC Clinician (4)* “… they gave us space at a [Tribal Health] office until our larger clinic was built … we created our office there and we were there for about two years. They didn’t renew the lease last fall and it was because they had begun to grow. So our challenge now is to stay connected because we’re about 25 miles apart now. So it’s different when you’re in the building right there with Tribal Health versus now being in a separate VA building a ways.” *HBPC Staff (8)*
	Ad hoc patient centered care	“[THP] provides primary care…more or less jointly with us, depending on the needs and desires of the patient. In some cases it may be a little bit more Home Based Primary Care doing that. In some cases it may be more [THP]…” HBPC Staff (2)
Patient Needs and Resources	Ad hoc patient centered care	“We look at, is there copays from the VA or not? Can we get the medications cheaper for them and have them directly mailed to their homes? So we really try to look at all of that. How can we save them on expenses as well as their healthcare?” *HBPC Staff (9)*
	Differences in VA and IHS/THP policy	“They receive free services from the Health Center and they don’t have any co-pays. So, it was a barrier for medications and other things that VA does have co-pays.” *Leadership (4)*
External policy	Differences in VA and IHS/THP policy	“It’s very hard to tell a [Native American] Veteran, “The VA’s going to charge you for this.” …because they don’t have to be charged in their system. So that’s a hindrance to recruiting some of our Native American Veterans, in that they have to pay for those services.” *HBPC Staff (3)*
		“The problem has been that we’ve gotten several referrals where we would have gladly provided the service, but the Veteran would have had a copay for the VA. Well, if I’m [Tribe B] and I have never paid a copay in my life for any medical service, I generally don’t like doing that.” *HBPC Staff (2)*
		“We went into this with some assumptions. …that the people on the reservation would socioeconomically be of a certain level. And we were incredibly surprised. Because while that was true for the most part, interestingly enough the veterans, who were a very tiny subgroup, were not always meeting the means test for the VA, which we were not allowed to waive.” *Leadership (4)*
INNER SETTING: Networks and Communications	Difficulty working in rural areas	“The problem is that connectivity can be really slow and a problem. So it can take you longer to do your documentation. We haven’t had a printer up until, I think we just got it so it now works but we’re talking for a year and a half we haven’t been able to print from there.” *HBPC Staff (2)*
Implementation climate: compatibility	Value of HBPC	“The providers in primary care have learned that if you’re having a problem trying to coordinate care in what’s happening to this patient, well, just get them enrolled in HBPC and it’ll happen magically. It isn’t real magic. It’s actually a lot of work. But that’s fine. I don’t mind that that’s part of our job, because it’s important.” *HBPC Staff (9)* “We see now a number of Native American veterans… [whose] lives … we are affecting, changing, making better. Changing their quality of life. You know, to me, that is value. “*Leadership (12)*
INDIVIDUALS: Knowledge and beliefs	Value of HBPC	“So the program itself is a huge benefit to everybody…because they’re so highly rural up there … our program can help them access the services to which they might otherwise not be able to access.” *HBPC Clinician (9)* “[The added value] for us it’s the variety of patients. For them, I think they get good care and some coordinated care within the realm of what they want.” *HBPC Clinician (*3) “I don’t know that anyone would have taken care of some of the people we take care of if we weren’t willing to kind of step out there a little bit.” *Leadership (1)*
	Value of working with new population to VA	“Our involvement with our Native American population has been a blessing to us… The fact that they allow us into their centers and their lives has, I think, enlightened and benefited everybody who works here in this HBPC program. …. So we are honored that they allow us to do this.” *HBPC Staff (1)* The last few years this project has kind of taken hold of my heart. I’ve met such great people and learned so much that it is important to me. *HBPC Staff (9)*
Other personal attributes	Experienced working with Tribes, IHS/THP	“I think having the inroads, having somebody familiar with the people there and somebody that the people there trusted I think made a lot of difference…” *HBPC Clinician (3)* “We couldn’t have done it without [an experienced American Indian health advocate] leading the way. Since she came to us from IHS, since she lived on the [local Tribe’s] Reservation…. They knew her already. They accepted her into their homes. And she was able to help convince them to accept the rest of us into their homes. So really, we couldn’t have done this without her. And we hadn’t done it prior to this.” *HBPC Staff (12)*
	Learning to work with Tribes IHS/THP	“Part of our goals that very first year was to become familiar with the system, to try to find a way to be able to address the leadership in the community. [A Tribal member] has been my liaison for the tribe since about day one. And has been just integral in helping me figure out what I need to do in a way that was respectful to the culture. So as a result, one of the things that I do every year with him is I go to all or most of the community clubs on the reservation… Because what we want to do is keep showing up in the different communities to let folks know that we’re really there, we want to continue to be there. As a result of that, a lot of things have really happened. One has been that there has been a slow acceptance of our members on the reservation and people have begun to recognize those folks.” *HBPC Staff (2)* “And we’re starting to see the fruits of [getting veterans signed up for benefits], in that people are coming up to us and thanking us for what we’ve done in that respect to help them. Takes a lot of time to do that process. And that’s extra. Me as a provider, that’s not counted on me seeing a patient and all that, doing that extra stuff for these veterans. It makes a difference.” *HBPC Staff (3)* “We spent a lot of time talking …, listening. And I think after several meetings where we really made it clear that we wanted to have an official relationship, we wanted to provide the kind of care that they wanted, that we wanted to be involved in their community, we got invited to a powwow, those of us that were reaching out. …. So I think showing that we were willing to step out of our comfort zone and go to them and do things within their culture really helped them to accept us as we started moving forward.” *Leadership (1)* “We do go to gatherings and represent the VA … especially when there’s a gathering American Indian Veterans. We …set up a little booth and we hand out flyers. And even on weekend or at night. We really try to be a positive presence at meetings. And we’ve had more people starting to stop by. First year there was almost nobody, and the last time we had more people, so that was nice. So I think getting out there and getting invited to community events is really important. “*HBPC Staff (12)*
PROCESS: Champions	Experienced working with Tribes, IHS/THP	My role is liaison in some ways between VA and the tribe, that’s kind of a grassroots level. …And so word gets around it’s a small community … I’m someone they know. And so I introduced the program to the community, letting them know we would be coming in and standing up this new project and kind of what our boundaries were.” *HBPC Staff (11)*
	Collaboration between VA and IHS/THP	“Many of the IHS staff I knew from before because I worked at Indian Health Service, so I knew a little how to negotiate their system.” *HBPC Clinician (12)*
Executing		“If IHS identifies somebody that’s having problems getting to a clinic or the Veterans’ Service Officer, the Tribal Veterans’ Service Officer can identify somebody with some transportation issues, health issues, any of those sorts of concerns that would make in-home health care advisable, then we’ll hear about it either from IHS or the VSO or sometimes the providers here in [Site I] or the CBOC, you know, if they recognize a need for home based we’ll get a referral.” *HBPC Clinician (7)* “Our referrals came directly from primary care at Tribal Health. So we tried to integrate ourselves by attending their meetings, giving presentations and just by physically being in their building, helped precipitate referrals. And then we attended their health fairs and a lot of veterans came up to our table that attended Tribal Health and also became our patients too. So there was a lot of working back and forth together in terms of health care.” *HBPC Staff (8)*
	Ad hoc patient centered care	“Usually referrals come from families, word of mouth. Somebody will say, “Hey, I know so-and-so. You might want to contact him,” or something like that.” *HBPC Clinician (7)* “…there has been a slow acceptance of our members on the reservation and people have begun to recognize those folks. And the other is that it is not uncommon for me to get a call from a family member or somebody that is caring for or involved with someone who needs our services to say, “What about Mr. So-and-so? Can you help him get enrolled in the system or figure out if he’s eligible for your service?” So I think that’s really one of the backbone pieces of how we’ve gotten to where we are.” *HBPC Staff (2)*
Reflecting	Image of VA	“But I think the path has been really increasing the positive image of the VA on the reservation and with the population. When we first went out there, there was a lot of reluctance from people in terms of letting us come in, especially those of us who were non-Native, with being able to come into their homes. And I think we’ve really found that that resistance has lessened pretty significantly over the last year or so, so that initial period with a little tough to convince people to let us in. They were waiting and seeing and making sure that we were still going to be around. And we don’t really have to sell the program like we used to, so I think that’s helping. We’re still expensive in terms of staffing and vehicle costs, certainly, but I think there are some intangible benefits that are certainly paying off for us.” *HBPC Staff (12)*
		[HBPC has] really opened the doors to us, in a way, to start the conversation about the agreement with [the Tribe]. We also had kind of an outreach event at [the Tribe]…to provide outreach and information to tribal veterans and their families …I think the fact that the HBPC programs and [HBPC Staff] in particular had been on the reservation for a couple years by then, meeting with people, talking with people, kind of being the face of the VA, and being okay—that they were trustworthy and had this relationship—it very well might be that if that hadn’t started, we may not have gotten that invitation to go there. “*Leadership (9)* “Because veterans who met HBPC enrollment criteria were too high income for the VA medical benefit, I was told that basically I lied, I didn’t tell them about this. The truth is, it was not a big highlight of our presentations over the preparations and months when we went into this. We, again, falsely assumed that this would not even come up. And we don’t own it. It’s a bigger VA regulation… I mean, talk about the mistrust, the miscalculation. I don’t think I ever really recovered completely from that. They still remind me of this.” *Leadership (4)*
	Building Relationship with Tribes, IHS/THP	“You know, just keep showing up. One of the things that [a Tribal member] told me in the beginning is that you can’t come out there and start a program and not keep showing up. If you really want this to work, you gotta keep showing up.” *HBPC Staff (2)* “Build relationships with both the Tribe and Indian Health Service because those are the folks that you really have to communicate with to keep all the resources flowing back and forth. Open communication is really important….They need to have input, and a stakeholder meeting before you start any program…and they can decide if they want to participate or not from day one.” *Leadership (12)* “…Make the Tribe or Tribes part of your planning process, get them involved in the planning and to define … catchment areas,…how many potential patients, a better demographic study…is the IHS facility aware that we are coming…what’s the process for getting them referred into our program and really have somebody that’s out front [as a point of contact].” *Project Staff (1)* “… every tribe is different. Every tribal leadership is different. The biggest thing is trust. And what you’re doing with a tribal organization is, they want to see you and they want to see you more than once. They want to see what you’re going to bring to them and what benefit they’re going to receive out of it. And they want to know you’re going to be there. … And so they want to make sure that it can be sustained. And they want to do it their way, too. “*Leadership (12)* The difficulty of establishing relationships with Tribal services [has] been a bit of a stumbling block. Although I think the fact that we’ve now kind of bypassed that by making our own relationships with veterans and have increased our profile on the reservation and have a more positive reputation is helping to alleviate that barrier a little bit.” *HBPC Staff (11)* “[This] is really a pretty small tribe… so… the numbers of folks that we have served ….are really small … It really is a relationship and the development of that relationship. And what I mean by that is trust. And our continued presence in that community. I think that’s why we are being successful. And having [VA staff] over there on the reservation, in the hospital forever, has been really helpful as well.” *HBPC Staff (2)*
	Opportunities for expansion	“But my idea of what would be ideal … [is] a full-time liaison that can work with the VA and IHS. And it would be a tremendous benefit if that person were Native and if the person were an RN. Because I can see this person working with all of the CHRs, all of the IHS providers, communicating directly …the VA provider—to the IHS provider. Kind of like the go-between” *HBPC Clinician (10)* “Have expanded contract services. Because there are agencies out there that are willing to go to these remote areas.” *HBPC Clinician (10)* “I would like to establish telehealth with tribal centers… [so when] issues occur with the Native American population that we can immediately respond.” *HBPC Staff (2)*

### Domain: intervention

#### Complexity

Expansion activities added complexity to the management of established HBPC programs. For most programs, a major bottleneck to implementing the intervention occurred when programs needed to recruit and hire additional nurse practitioners as primary care providers. Delays in hiring processes also contributed to postponing initiation of some rural HBPC programs. Efforts to reduce the typical difficulty in recruiting and retaining primary care providers to rural areas by adding recruitment financial incentives to attract candidates were not always successful.

Other challenges for management and service delivery were related to the rural setting. Driving long distances to see patients in their homes was commonly referred to as “windshield time.” Travel could be challenging in inclement weather and with poor road conditions. The isolation of some reservation communities and difficulty in locating patients contributed to travel times, which might involve several hours per patient. Some programs reduced caseloads to account for the impact of travel time on scheduling and providing home care. In remote areas, faulty or poor connectivity hampered communications by cell phones or internet, leading to delays in communicating with patients and HBPC team members, as well as to accessing patient electronic health records.

Another layer of complexity was adopting a new process for referring potential HBPC patients for admission screening. Unlike HBPC programs at the VAMC where patients are referred by their VA primary care providers, these expansion programs often engaged in outreach activities to identify potential patients many of whom were not yet enrolled for the VA medical benefit. This was not a normal part of HBPC activities. A common solution was participation by a VA benefits officer, along with HBPC staff, at Tribal health fairs or social gatherings to explain VA medical, financial, and burial benefits and to expedite enrollment. HBPC staff also promoted their programs directly to Tribal community members at health fairs, community meetings (e.g., Veterans of Foreign Wars), and events (e.g., pow wows).

#### Cost

Respondents attributed grant funding from the VA Office of Rural Health as the sole rationale for VAMC to initiate rural expansion programs; some added that the decision was based on the expectation of future financial sustainment through the annual funding allocations. The proportion of Native and non-Native veterans varied across programs from an estimated 20 to 50%, with an average daily census of 1-25 veterans. Because American Indian populations are relatively small, program sustainment also depended on expanding HBPC to non-Indian rural-dwelling veterans to justify and recoup costs in subsequent years’ funding allocation. Another concern about sustainment was balancing staffing and program growth. Programs that assigned existing personnel to rural HBPC as collateral duties might need to limit capacity if there were no additional future personnel hires.

### Domain: outer setting

#### Cosmopolitanism

Local contexts were particularly evident in the degree of networking between VA and IHS/THP. For instance, one VAMC successfully arranged for joint privileging of several VA and THP staff, while another VAMC was advised against such an arrangement by its regional legal advisors. Some programs insisted that VA be the provider of record while other programs allowed for patient choice between VA and IHS. Coordinated case management with IHS/THP also varied from fully integrated, to simple notification of recommendations, to no formal coordination. Having a previous working relationship between a VAMC and IHS/THP or Tribe did not guarantee acceptance of the previously unknown HBPC service or its requirement to deliver care in patients’ homes on reservations.

Networking between VA and IHS/THP clinicians was advantageous to programs and patients. Availability of local IHS/THP services allowed HBPC care managers to expand the range of resources to meet patients’ needs and preferences. For instance, where VA physical therapy services were not available in distant remote communities, patients might receive care locally through IHS/THP. Likewise, IHS/THP care managers were able to expand the range of resources, such as durable medical equipment, through the VA. Clinical coordination occurred on an ad hoc basis. Co-location of VA and IHS clinics facilitated relationships between providers but if HBPC moved to another site, that past advantage diminished.

Other formal and informal co-management strategies were also found in multiple cases, VA prescriptions were accepted at IHS/THP pharmacies. Availability through IHS/THP was advantageous for patients without residential mail delivery because the VA does not send medications to post office boxes. It was also advantageous for many patients because there is no co-pay requirement if medications were dispensed by IHS/THP. Several programs also developed processes to work with an IHS/THP partner to identify appropriate patients by direct phone call to a HBPC point of contact, a standardized referral form, or shared electronic health record.

#### Patient needs and resources

Patients’ needs are identified through a standardized HBPC screening process before admission to the program. Once admitted to the program, patients have access to comprehensive interdisciplinary primary care as well as referrals to other specialized VA services. In addition, the importance of the IHS/THP resource was a recurring theme in managing patient-centered care with resources that might be locally available from IHS/THP and at a reduced cost to the patient.

#### External policies and incentives

The VA-IHS Memorandum of Understanding supported collaborations but seamless and effective co-management and communication about mutual patients was stymied in most programs by the lack of a shared electronic health record. Even where joint privileging occurred, current federal policy prevents records from being interoperable; providers and staff entered patient information separately into both VA and IHS/THP records. At the patient level, seamless co-management was impacted by VA policy requiring co-pays depending on priority group, which sharply contrasted with IHS/THP policy to provide healthcare at no charge. These unexpected policy differences may have been a disincentive to patient enrollment for the VA medical benefit that might require co-pays for VA medical services and medications.

The direct incentive to initiate expansion was financial grant support. Indirect incentives included oversight from mandatory reporting requirements for HBPC and progress reports for the expansion grant; these were monitored by VAMC and regional managers as well as by the VA Offices of Rural Health and Geriatrics/Extended Care. VA provided no centralized guidance on how to conduct expansion but convened voluntary monthly telephone conferences for about a year that allowed programs to share experiences.

### Domain: inner setting

#### Structural characteristics

HBPC is a mature program with well-defined structure and quality performance standards that allow flexibility to operate within local conditions (e.g., drive times, coverage area, patient complexity, staff turnover). The program goal remained unchanged but access for all rural-dwelling veterans was limited by staff size and round-trip distance to patients’ homes.

#### Networks and communication

The internal VA electronic health record and availability of Virtual Private Networks and cellphones to allow electronic health record access were assets. However, use of these technologies was often challenged by poor cell phone reception or inadequate internet connectivity in rural areas, as well as lack of networked office equipment at VA, IHS/THP, government, or non-government facilities where office space was assigned to HBPC staff.

#### Implementation climate: compatibility

HBPC programs regularly receive admission referrals from VA primary care clinics. One respondent recalled a colleague’s remark describing HBPC as “magic” because of the program’s success in managing complex patients. Weekly interdisciplinary team meetings to address the comprehensive needs of patients also functioned to support teamwork regardless of staff physical location or catchment areas. These regular team interactions became opportunities for peer-to-peer education. Nearly every program identified the team-based case conferences as sources of cultural competency education for working with American Indian veterans. Organizationally, HBPC is also recognized as a valuable fiscal asset to VAMCs through the VA annual funding formula.

### Domain: individuals

#### Knowledge and beliefs

Many key clinical and administrative personnel were knowledgeable about HBPC approach and goals because they were expanding an existing clinical program. Several clinicians noted, however, that they had been unfamiliar with the requirements for VA benefits (i.e., medical, burial, and financial) when they started outreach efforts on reservations and tried to expedite enrollment for the VA medical benefits for potential HBPC users and other Native community members. Overall, HBPC staff perceived the intervention as positive, both to communities and to veterans who had previously been under-served.

#### Other personal attributes

Most expansion programs were facilitated by clinical and coordination personnel that had gained culturally appropriate experience from being a tribal member or from previous employment with IHS/THP. Their familiarity with American Indian customs and their culturally sensitive interactions with Tribes, IHS/THP, and Native communities promoted acceptance of HBPC. Without this background, staff and clinicians were also successful in promoting HBPC if they accepted the guidance of community members, including participation in community events and meetings and visiting communities repeatedly to build trust and acceptance. These personalized relationships were beyond the scope of the typical clinical visit and were valued by both the Native community and by the HBPC personnel.

### Domain: process

#### Planning

At each VAMC, planning focused on internal processes of expanding staff or catchment area. Population-based needs assessments were not conducted to estimate the potential number of American Indian HBPC-users prior to implementation. A common, but erroneous, assumption was that all of veterans in the catchment area would be eligible for the VA medical benefit. Although VA and/or IHS/THP staff identified potential HBPC users, some of these vulnerable patients were turned away after failing to meet the financial criterion for medical benefits eligibility. As a result, it may have appeared that VA was not delivering the promised care to veterans who were in need.

#### Champions

Clear VA champions emerged in implementation of HBPC in the roles of the program coordinators or clinicians who were able to establish relationships with American Indian communities as well as with IHS/THP. Most respondents easily identified the one individual who had the most impact for their respective programs and cited the personal attributes as a reason for that individual’s success. In some locales, kick-off activities may also have benefitted from the direct involvement of leadership at the facility, regional administration, or headquarters level to initiate introductions and promote HBPC with a Tribe or IHS/THP clinic.

#### Executing

Overall, program implementation took longer than anticipated and was facilitated by champions who emerged in American Indian communities. Community Veteran advocates assisted HBPC expansion by introducing the program leadership at community meetings; over time, the program became accepted and its value embraced by the community. In addition to the usual internal VAMC referral process, referrals to HBPC were made on an ad hoc basis from IHS/THP clinicians, Veterans Service Officers, Tribal Veteran Representatives, and other Tribal health and service departments. Family members began reaching out to HBPC on behalf of their loved ones. Several HBPC programs developed unique direct referral options for IHS/THP using a FAX version of the VAMC standardized referral form, electronic health record referral process, or telephone call to a VA point of contact.

#### Reflecting

HBPC improved the image of VA in most communities by promising a service and delivering on that promise. HBPC personnel’s frequent visits for patient care, for outreach and to participate in community events established personal relationships of acceptance and greater trust for the VA. Positive experiences were associated with successful formal or informal efforts to integrate clinical collaborations between VA and IHS/THP.

All interviews ended with a query about recommendations to other VAMC based on lessons learned from the implementation experiences. Common responses were about building relationships with both the Tribe and IHS/THP, recognizing their roles and inputs as stakeholders and allowing them to define their level of participation. Joint planning efforts would also be valuable to define the catchment area, estimate the potential population, and determine if there are gaps in care. Expansion of VA services was also envisioned through telehealth collaborations with IHS/THP, better clinical integration through shared medical or pharmacy records, identifying space for HBPC staff in American Indian communities or IHS/THP buildings, and executing contracts with Tribal and non-Indian home health agencies that might operate in areas too distant for HBPC to regularly visit. Finally, recommendations also included awareness of practical issues, such as adapting to the distances involved in rural areas and training HBPC staff in local cultures and about the VA medical benefit.

## Discussion

This study begins to fill gaps in the literature on implementing HBPC in rural areas, as well as developing clinical programs in coordination with IHS/THP. CFIR was a useful instrument to systematically organize data and identify shared issued across sites. Despite variation in local contexts, there was consistency in experiences that might inform planning to expand access for medical services for populations in remote rural communities and to coordinate services among healthcare providers. Although HBPC is a standard benefit with centrally authorized guidelines, implementation in rural areas added complexity to intervention’s existing program structures and processes. Key personnel facilitated successful expansion programs through their personal interactions to with Tribes, IHS, and community members. These individuals were not in management leadership positions but represented the HBPC program and, consequently, the VA. In the process of implementation, champions arose in both the VA and American Indian communities, underscoring the significance for this population of developing personal relationships to establish trust and acceptance of new programs [42]. External policies promoted expansion of a well-established urban program but allowed local programs the flexibility to manage the practical aspects of coordinating care with other healthcare organizations and other government entities. Flaws in the process were noted by respondents, including the lack of a population-based needs assessment, planning in coordination with IHS/THP, as well as differing policies for medical benefits and for inter-agency communications. Those problems indicate potential conflicts between outer and inner settings in implementing programs into underserved communities.

Several challenges and barriers were identified, some that could not be overcome. Difficulty in recruiting staff and delays inherent in the VA hiring process led to postponing programs, limiting program growth, or developing new program models. The distance to the most remote areas of a reservation may continue to be an obstacle to full access for American Indian and Alaska Native veterans. With relatively small numbers of American Indian veterans admitted to HBPC, sustainability over time depends on the average daily census of rural-dwelling non-Indian patients. Finally, differing VA and IHS policies on eligibility for medical benefits and schedules of cost for services were significant external barriers. The VA medical benefit income test resulted in turning away American Indian veterans who may have met HBPC admission criteria or who opted out of using HBPC when co-payment was required.

This study has a number of limitations. Respondents were selected for knowledge of the program but may have lacked overall background knowledge about the VAMC relationships with Tribes and may not have been fully aware of federal and Tribal policies. Interviews took place after the original grant-funding period and memories may have faded. Data collection was limited to interviews and no other source of information (e.g., diaries, meeting minutes) was available.

A goal of qualitative research is to document the range of variation and identify common experiences rather than a statistical description of variation and, therefore, the results may be limited to these specific case studies. The USA is unique in that American Indian and Alaska Native communities are considered sovereign nations under the US Constitution. Although other countries do not extend right of sovereignty to Native populations (e.g., Canadian First Nations and Australian Aborigines), the study may be applicable to expanding access and coordinating healthcare in discrete ethnic communities. The scope of the study is also limited by its focus on developing a non-institutional long-term care service for populations that may be served by other providers of record. However, the need to collaborate across healthcare organizations is also a unique contribution of this study. The literature on HBPC has not previously addressed how healthcare organizations reach out beyond their own patient populations to expand access. Finally, since there is no comparative literature on implementation of HBPC in rural areas [1], corroboration of our findings requires further research to understand the extent to which these barriers and facilitators might apply to other rural communities.

The context for expansion of access to deliver non-institutional long-term care was the policy agreement detailed in the VA-IHS Memorandum of Understanding of 2010. Overall, HBPC expansion efforts were successful although the target populations were relatively small. Where enrollment for VA benefits was uncommon in some communities, HBPC case finding efforts were linked to identifying and enrolling veterans for medical and other benefits. The HBPC Handbook places the responsibility for all aspects of management, planning, and developing community relationships on the HBPC program director. Thus, the expansion and innovation experiences might not be widely shared through the VAMC or regional levels unless a national level effort is undertaken.

## Conclusion

Opportunities for shared learning would benefit federal healthcare organizations to expand other medical services to remote rural areas, American Indian reservations, and other underserved communities. Planning efforts should take into account conducting a population-based needs assessment and allowing sufficient time to develop trusting relationships with Tribes, IHS/THP, and Native or other underserved communities. Bottlenecks and delays in the hiring processes should also be considered in determining if the rollout will be phased or the program implemented at full capacity. Planning efforts should also consider availability of IHS/THP resources for patients and develop opportunities for co-management to prevent unintended duplication of effort, over-prescribing of medications, and other inefficiencies. For the VA, these planning and coordination activities may become increasingly important as VAMC enter into Reimbursement Agreements for primary care services provided by IHS/THP to veterans and as VA continues to expand HBPC to other rural areas.
